# Phytochemical Composition and Antioxidant Activities of *Sonneratia caseolaris* (L.) Engl. Leaves and Roots: Insights Into a Promising Mangrove Species

**DOI:** 10.1002/fsn3.71664

**Published:** 2026-04-18

**Authors:** Federico Cerri, Stefania Pagliari, Shazla Mohamed, Massimo Labra, Luca Campone, Paolo Galli

**Affiliations:** ^1^ Department of Earth and Environmental Sciences DISAT University of Milano—Bicocca Milano Italy; ^2^ MaRHE Centre (Marine Research and Higher Education Center) Magoodhoo Island Maldives; ^3^ Department of Biotechnology and Biosciences University of Milano—Bicocca Milano Italy; ^4^ The Maldives National University Malé Maldives

**Keywords:** antioxidant activity, mangrove natural products, metabolome analysis, pharmacological potential, polyphenolic compounds, UPLC‐ESI/HRMS

## Abstract

Mangroves thrive in extreme environments and produce secondary metabolites with significant pharmacological potential, making them a rich reservoir of bioactive natural products for the development of new therapeutic agents. *Sonneratia caseolaris* (mangrove apple) has long been utilized in traditional medicine for its antioxidant, antimicrobial, and antifungal activities, although comprehensive investigations of its phytochemistry and biological activities remain limited. In this context, the present study provides the first comprehensive profiling of the chemical constituents and antioxidant capacity of *S. caseolaris* leaves and roots collected in the Maldives. Ethanol 50% extracts of leaves and roots were analyzed using ultraperformance liquid chromatography‐electrospray ionization‐high resolution mass spectrometry (UPLC‐ESI/HRMS) operated in positive and negative ionization modes and antioxidant potential was evaluated by spectrophotometric ABTS, DPPH, and ORAC assays. A total of 45 molecules were detected, predominantly polyphenols, including phenolic acids, flavonoids, and tannins. Flavonoid glycosides and gallotannins were the major groups, with several compounds not previously documented in this species or among mangroves. Both extracts exhibited strong antioxidant responses, yielding IC_50_ values comparable to or lower than ascorbic acid. These findings expand the phytochemical knowledge of *S. caseolaris*, underscore its antioxidant activity and highlight its pharmacological and nutraceutical potential.

## Graphical abstract

UPLC‐ESI/HRMS profiling of the mangrove species *Sonneratia caseolaris* identified 45 metabolites in leaves and roots, mainly flavonoid glycosides, gallotannins, and other polyphenols, including two sulfated flavonoids not previously reported in mangroves. Most compounds are newly identified for this species, suggesting that the unique Maldivian habitat may shape its distinctive metabolite profile. Both extracts showed strong antioxidant activity (ABTS, DPPH, ORAC), highlighting *S. caseolaris* as a promising source of nutraceutical and pharmacological phytochemicals.

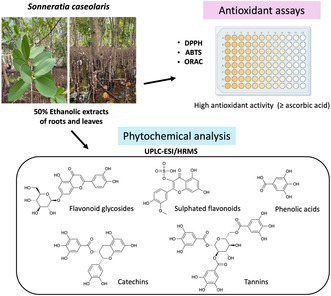

## 1 Introduction

Plants, as sessile organisms, have evolved complex biochemical systems that enable them to withstand a range of environmental pressures. These systems often involved the production of a wide range of secondary metabolites that contribute significantly to the defense mechanisms and adaptation of plants (Teoh 2016). Over time, such compounds have proven valuable in the search for new therapeutic agents (Rates 2001; Sharma et al. 2019; Wal et al. 2025). Despite major advances in synthetic chemistry, natural products and their derivatives still account for one‐third of all Food and Drug Administration (FDA)‐approved new molecular entities (NMEs) (Patridge et al. 2016), and more than half of approved pharmaceuticals between 1981 and 2010 have been natural products, their derivatives, or molecules inspired by natural scaffolds (Newman and Cragg 2016; Davis and Choisy 2024). Plant‐derived metabolites are particularly prominent within this group, with approximately 25% of approved drugs worldwide originating directly or indirectly from plant sources (Davis and Choisy 2024).

Among the various plant groups, mangroves are particularly noteworthy for their biochemical diversity, which is shaped by their genetic richness and the challenging ecological conditions of their habitats. Their resilience to high temperatures, high salinity, tidal fluctuations, and other stressors is often mediated by unique bioactive compounds. Additionally, many mangrove species have been used in traditional medicine, further supporting their potential in drug discovery (Audah et al. 2022; Cerri et al. 2022). However, the exploration of mangroves for novel bioactive compounds remains relatively underdeveloped (Patra and Thatoi 2011).

The genus *Sonneratia*, within the Sonneratiaceae family, is distributed across the Indo‐West Pacific and has gained increasing interest because of its potential pharmacological value. However, detailed investigations into the chemical composition and bioactivity of these taxa are still limited (Wu et al. 2009). The genus includes six recognized species along with several interspecific hybrids (Zhou et al. 2010). Of these, *Sonneratia caseolaris* (L.) Engl., commonly referred to as the mangrove apple, is widely found in countries such as the Maldives, Sri Lanka, China, Bangladesh, the Malay Peninsula, Indonesia, Borneo, the Philippines, Timor, New Guinea, the Solomon Islands, and northern Australia (Cerri et al. 2024; Dev et al. 2021).

*S. caseolaris* has traditionally been employed to treat a variety of ailments, including infections, inflammation, gastrointestinal disorders, and skin conditions and is reputed for its antioxidant, antimicrobial, and antifungal properties (Dev et al. 2021; Bandaranayake 1998). Scientific interest in this species has grown in recent years, with several studies identifying bioactive compounds. In particular, leaf extracts have been shown to represent a rich source of metabolites contributing to its biological activities, including phenolic acids such as gallic, ellagic, and vanillic acids, hydroxycinnamic acids, flavonoid aglycones (e.g., luteolin, quercetin, apigenin, myricetin) and flavonoid glycosides, catechins, phytosterols, fatty aldehydes, and fatty alcohols (Audah et al. 2022; Kundu et al. 2022; Khanh Tran et al. 2023; Cerri and Galli 2025). Nevertheless, the majority of this research has been geographically limited to countries such as India, Vietnam, Bangladesh, and China.

Given the influence of geographical and environmental conditions on plant phytochemistry (Khanh Tran et al. 2023; Jin et al. 2009), it is important to study this species in other less‐studied regions. In this context, the present study examines the phytochemical constituents and antioxidant potential of *S. caseolaris* leaves and roots collected from the Maldives, a region with unique ecological characteristics that may affect metabolite production. The chemical profiles of both organs were characterized by coupling ultra‐performance liquid chromatography with high‐resolution mass spectrometry (UPLC‐HRMS). Notably, this study constitutes the first in‐depth chemical analysis of the roots of *S. caseolaris*. Furthermore, various in vitro assays were conducted to evaluate the antioxidant efficacy of the extracts.

This study contributes valuable insights into the phytochemistry of *S. caseolaris* and reinforces the potential of mangrove species in the development of future therapeutic and nutraceutical applications.

## 2 Experimental

### 2.1 Plant Sources and Collection

**FIGURE 1:**
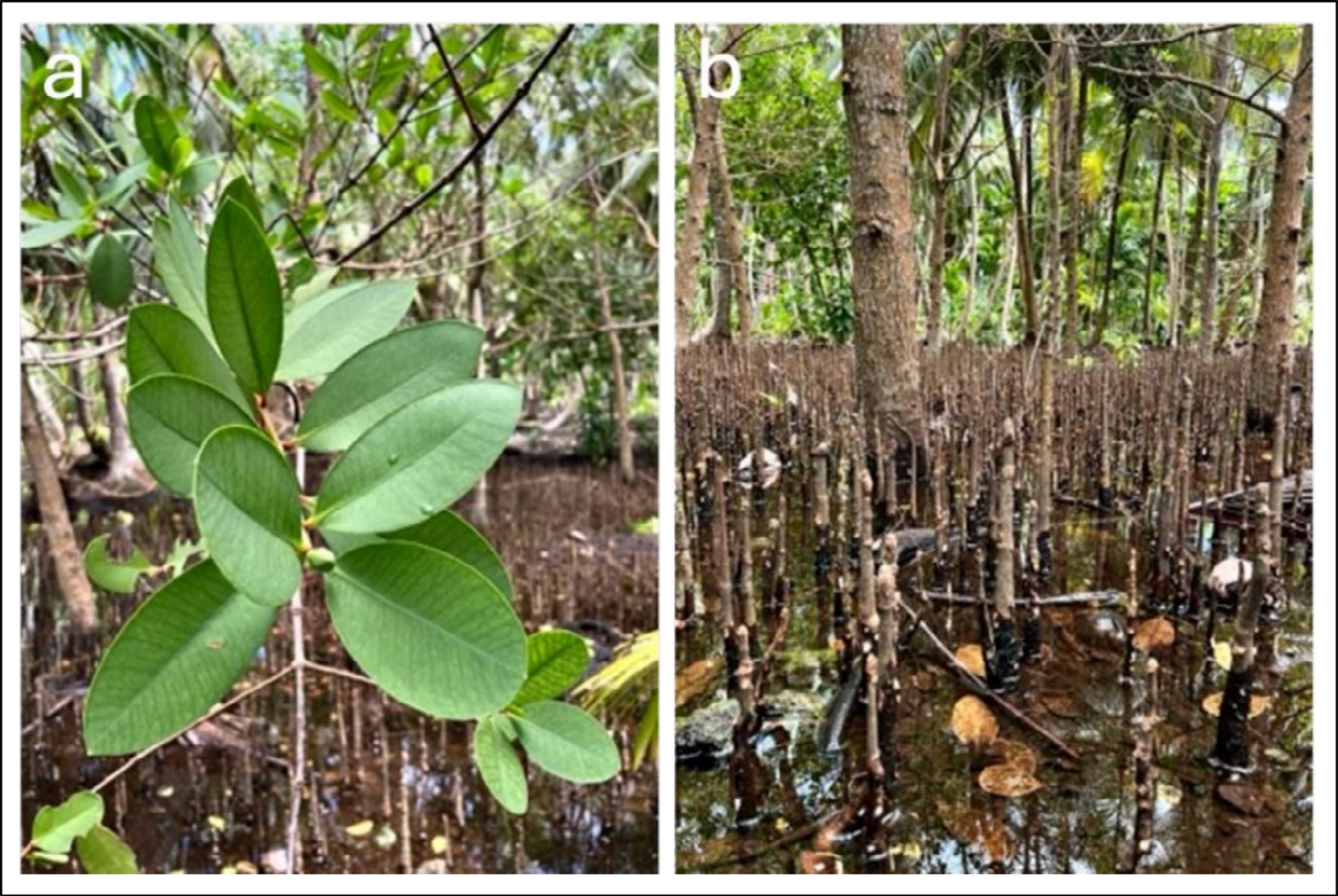
Leaves (a) and roots (b) of *S. caseolaris*.

Leaves and roots of *S. caseolaris* were gathered from at least ten adult, healthy individuals located in Baa Atoll, Maldives 5°17′50.0″N, 72°58′06.0″E during the month of November 2024, at the end of the wet season (Figure 1). The identification of the species was based on morphological traits as described by Primavera et al. (2004). Since no fruits were available at the time of collection, they were excluded from the current study. The sampling site corresponds to a closed‐system, marsh‐based inland mangrove (Cerri et al. 2024), occupying a low‐lying depression with muddy substrate and shallow surface water. Water salinity was measured in situ at three points using a handheld multiparameter probe (Hanna Multiprobe Meter, model HI98494, Hanna Instruments Inc., USA), yielding a mean salinity value of 0.16 ± 0.07 PSU. After harvesting, the samples were rinsed thoroughly with distilled water to eliminate surface contaminants.

### 2.2 Extraction Procedure

The samples underwent lyophilization and the dried material was then ground to a fine powder using a Grindomix GM 200 knife mill (Retsch, Haan, Germany). Ultrasound assisted extraction (UAE) was carried out to obtain the plant extracts. For each extraction, 0.5 g of dry sample (DW) was transferred to 50 mL polypropylene tubes and combined with 10 mL of either 50% ethanol‐water (v/v), pure ethanol, or pure water. Following manual mixing, the samples were sonicated in an ultrasonic bath (Sonorex Tk 52; Bandelin Electronic, Berlin, Germany) for 10 min at 25°C. After sonication, the mixtures were subjected to centrifugation at 6000 rpm for 5 min and the resulting supernatants were carefully collected. This step was performed three times per sample and the collected supernatants were mixed and filtered using Whatman Grade 0965 filter paper to eliminate fine particulates. Solvent removal was achieved with a Hei‐Vap Core rotary evaporator (Heidolph Instruments, Schwabach, Germany) and the residues were subsequently lyophilized. For the subsequent spectrophotometric analyses (ABTS, ORAC and DPPH), the extracts were initially solubilized in water or 50% ethanol to produce stock solutions with a concentration of 10 mg/mL. Operative concentrations were then obtained through dilution in water. The extracts were preserved at −20°C for later analysis.

### 2.3 Phytochemical Analysis of *Sonneratia caseolaris* by UPLC‐DAD‐HRMS/MS

Chemical analysis of the extracts was conducted using a UPLC/HRMS system (XEVO G2‐XS QTOF, WATERS CORP., MILFORD, MA, USA) and electrospray ionization operated in positive and negative ionization modes. Chromatographic separation employed a binary mobile phase: solvent A consisted of 0.1% formic acid in water and solvent B was 0.1% formic acid in methanol. The elution protocol began with 5% B for the first 2 min, increased linearly to 95% B over the next 14 min, held at 95% for 5 min, then returned to starting conditions for a 5‐min reequilibration. A flow rate of 400 μL/min, and an injection volume of 5 μL of 0.5 mg/mL sample (1 mg dissolved in 2 mL of 50% methanol), and a column temperature of 30°C were used. UV detection ranged from 210 to 400 nm.

Mass spectrometric settings included a 2.0 kV capillary voltage, source temperature of 150°C, and desolvation temperature of 500°C. The cone gas flow was set to 10 L/h and desolvation gas to 1000 L/h, with spectra acquired over *m/z* 50–1000. Data‐dependent MS/MS targeted the two most intense precursors per scan at 30 V collision energy. The compound annotation relied on the exact mass, retention time, UV–Vis profiles, and fragmentation patterns and was categorized using the Metabolomics Standards Initiative (MSI): Level 1: confirmed via comparison with authentic standards; Level 2: putative identification using MS^2^ library or literature data; Level 3: tentative classification based on structural similarity and chemotaxonomic inference.

### 2.4 Assessment of Antioxidant Capacity

#### 2.4.1 ABTS Assay

The 2,2′‐azino‐bis(3‐ethylbenzothiazoline‐6‐sulfonic acid) (ABTS) assay was conducted following the method of Pagliari et al. (2024) with some modifications. A 9500 μL aliquot of 0.1 mM ABTS solution was mixed with 50 μL phosphate‐buffered saline (PBS), standard or extracts (0.02–1.00 mg/mL). 300 μL of each mixture was pipetted into a 96‐well microplate and placed in the dark for 30 min. Absorbance readings were taken at 734 nm with a microplate reader (Infinity M Nano+. Tecan Italia Srl). Ascorbic acid (AA) served as reference standard and IC_50_ values (μg/mL) were determined using GraphPad Prism v.8, with all tests performed in triplicate (*n* = 3).

#### 2.4.2 DPPH Assay

The 2,2‐diphenyl‐1‐picrylhydrazyl (DPPH) assay was conducted following the method of Pagliari et al. (2022). 5 μL of the extract or standard (2.5–1000 μg/mL) was mixed with 950 μL of 0.1 M DPPH• solution. Following a 30‐min incubation at ambient temperature, absorbance readings were taken at 515 nm. AA served as reference standard IC_50_ values (μg/mL) were based on triplicate measurements (*n* = 3).

#### 2.4.3 ORAC Assay

The oxygen radical absorbance capacity (ORAC) assays were conducted in black 96‐well plates following Pagliari et al. (2024). Each well received 100 μL extract (aqueous) at 0.02 mg/mL, 100 μL of 590 mM 2,2‐Azobis (2‐methyl‐propionamidine) dihydrochloride (AAPH) in PBS (pH 7.5), 25 μL fluorescein, and 100 μL PBS. Fluorescence (excitation at 485 nm and emission at 530 nm) was recorded every 5 min over 1 h at 37°C. AA served as the reference standard. IC_50_ values (μg/mL) were based on triplicate measurements (*n* = 3).

### 2.5 Data Analysis and Statistics

Results are expressed as mean ± standard error of the mean (SEM) (*n* = 3). Antioxidant assay outcomes (ABTS, DPPH, ORAC) for leaves, roots, and controls were evaluated by one‐way ANOVA with Tukey's post hoc test, with *p* < 0.05 regarded as statistically significant.

## 3 Results and Discussion

### 3.1 Selection of Extraction Solvent

Mangroves are recognized for their abundant production of polyphenolic compounds, which are known for their strong antioxidant properties. These bioactive compounds can be efficiently extracted using green solvents such as ethanol‐water mixtures (Palaiogiannis et al. 2023; Huamán‐Castilla et al. 2024). Water and ethanol are commonly used to recover phenolic compounds as a result of their low toxicity and high extraction efficiency. Compared to other commonly used organic solvents for polyphenol extraction, such as methanol or acetone, ethanol is significantly less toxic and is generally recognized as safe (Generally Recognized As‐Safe according to the US Food and Drug Administration), making it a preferable choice for use in the food, nutraceutical and pharmaceutical industries (Galanakis 2012; Chaves et al. 2020). Furthermore, using aqueous ethanol reduces the environmental impact and risks associated with handling and disposing of solvents compared to using more aggressive organic solvents.

**FIGURE 2:**
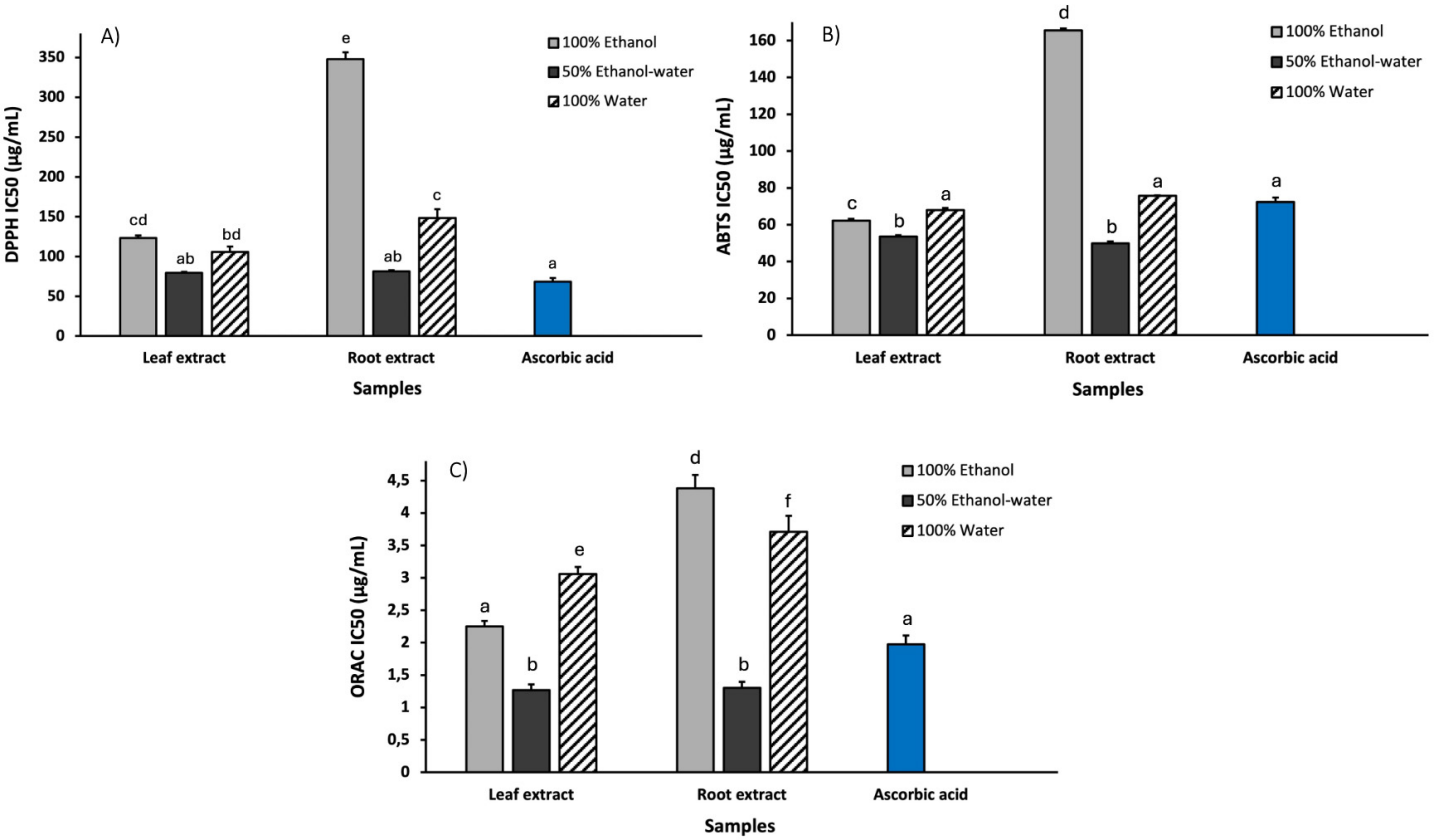
Antioxidant activities of *Sonneratia caseolaris* leaves and root extract determined by DPPH (A), ABTS (B), and ORAC (C) assays using different solvents (100% EtOH (gray), 100% water (lined), and 50% EtOH–water (black)). Ascorbic acid was used as a positive control (blue). Antioxidant capacity is reported as IC50, and all measurements were performed in triplicate (*n* = 3). Different letters in each graph indicate a statistical difference (*p*‐value < 0.05).

To identify the most appropriate extraction solvent, different solvent systems were compared considering extraction yield, metabolite coverage, and antioxidant activity. Accordingly, three solvents were tested for the extraction of secondary metabolites from *S. caseolaris* leaves and roots: pure ethanol, a 1:1 (v/v) ethanol–water mixture, and pure water. The extraction yield strongly depended on solvent composition. For *S. caseolaris* leaves, the highest yield was obtained using the 50% ethanol–water mixture (41.24%, 412.4 mg/g DW), which resulted in higher yields than both pure ethanol (26.20%, 262.0 mg/g DW) and water (31.76%, 317.6 mg/g DW). In contrast, root extracts showed lower and less variable yields across solvents, ranging from 16.26% to 21.92% (162.6–219.2 mg/g DW). Overall, the hydroalcoholic mixture proved to be the most efficient extraction solvent, particularly for leaf material. This trend is consistent with previous studies reporting optimal recovery of phenolic compounds at intermediate ethanol concentrations (around 50%) when using UAE (Paini et al. 2016). Indeed, since secondary metabolites such as polyphenols constitute a chemically heterogeneous class with varying degrees of hydrophilicity depending on the number of hydroxyl groups and molecular size, hydroalcoholic mixtures are particularly effective in offering a broader range of polarity than pure solvent solutions (Vijayalaxmi et al. 2015). To further compare the metabolic profiles of the extracts obtained with the three different solvents, HPLC–UV analysis was carried out at 280 and 330 nm, wavelengths typically associated with phenolic compounds. No significant differences in the chromatographic profile were observed for any of the extracts suggesting a similar qualitative extraction capability of the solvents (Figure S1). Finally, the antioxidant activity of the extracts obtained with the three solvent systems was also evaluated using spectrophotometric assays (DPPH, ABTS, and ORAC). These analyses showed that the 50% ethanol extract exhibited higher radical‐scavenging activity compared to the pure solvents (Figure 2). This effect was particularly evident for root extracts, where the 50% EtOH extract displayed significantly greater antioxidant activity than both water and ethanol extracts across all three assays (Figure 2A–C). Based on the combined evaluation of extraction yield, metabolite coverage, and antioxidant activity, the 50% ethanol extract was selected for subsequent characterization.

### 3.2 Characterization of Compounds From *Sonneratia caseolaris*

**FIGURE 3:**
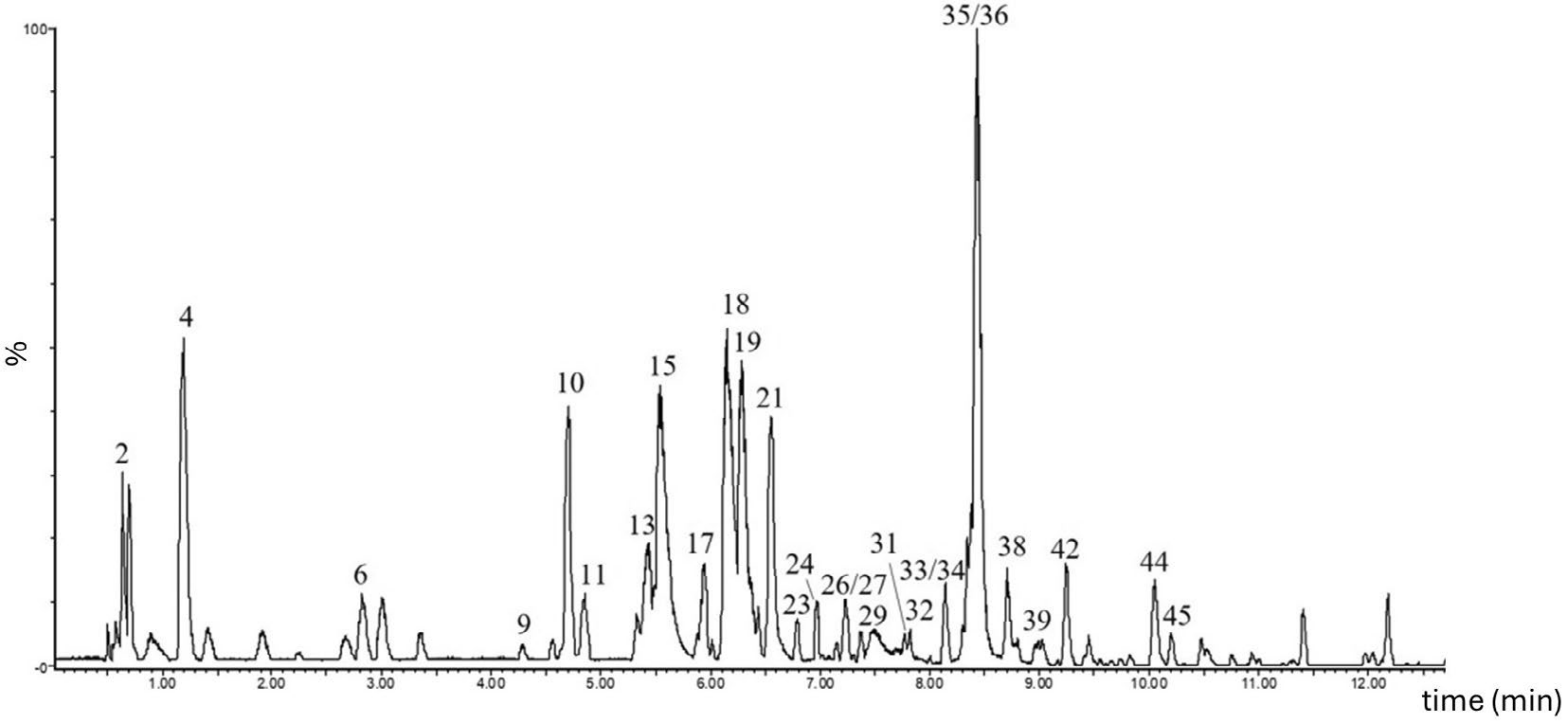
Chemical profile of *Sonneratia caseolaris* leaves: UPLC‐HRMS chromatogram in negative ion mode (*m/z* 50–1200). The numbered peaks correspond to the compounds listed in Table 1.

**FIGURE 4:**
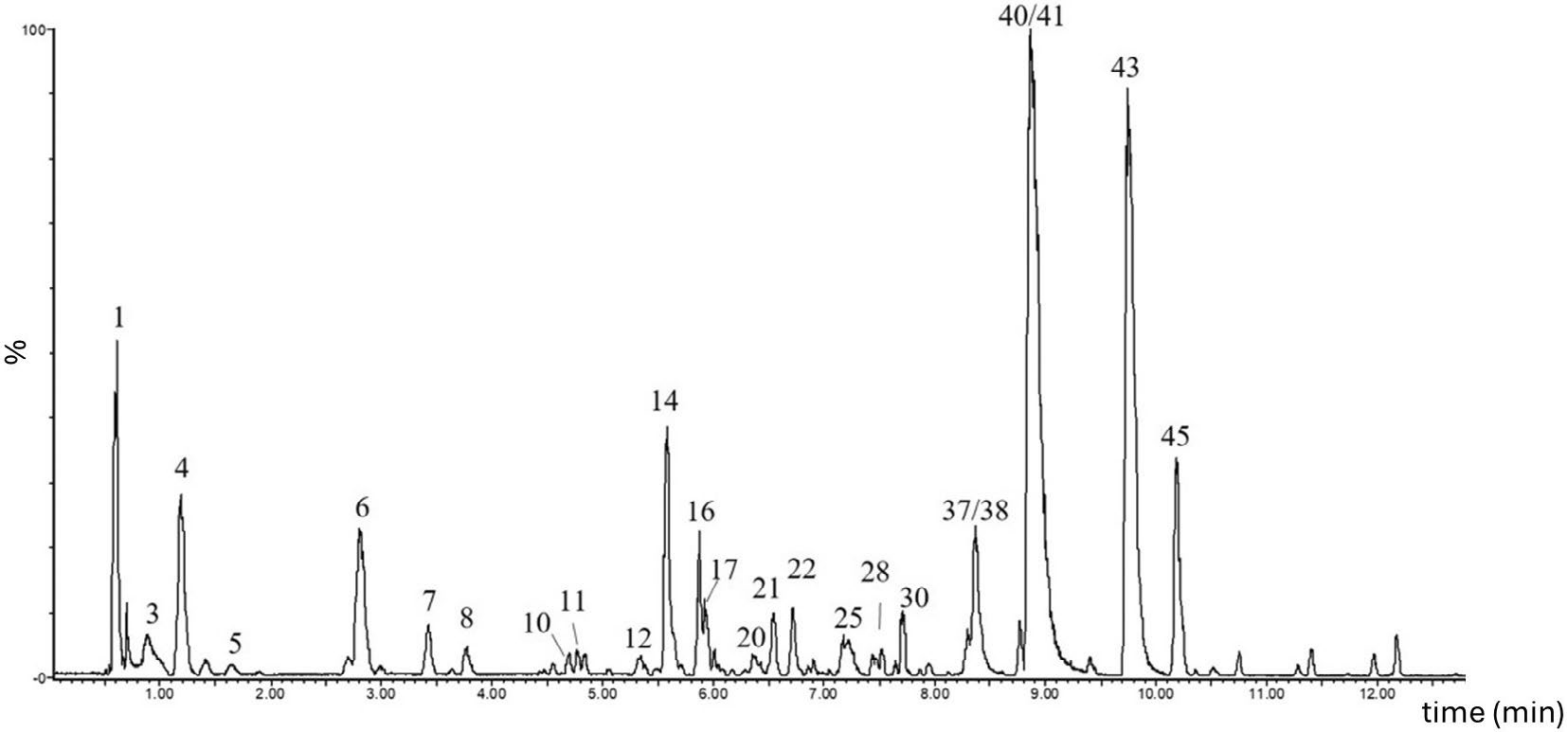
Chemical profile of *Sonneratia caseolaris* root: UPLC‐HRMS chromatogram in negative ion mode (*m/z* 50–1200). The numbered peaks correspond to the compounds listed in Table 1.

Following the selection of the most suitable extraction solvent, a detailed chemical characterization using high resolution mass spectrometry analysis of *S. caseolaris* leaves and roots extracts (EtOH 50%) was carried out. Analyses were performed using both positive and negative ionization modes to obtain complementary information on the metabolite profiles. The chromatographic profiles of leaves and roots are presented in Figures 3 and 4 respectively. A total of 45 compounds were detected, of which 20 were exclusive to the leaves, 17 to the roots, and 8 were common to both extracts (Table 1). Most of the identified molecules belong to the polyphenols group. Polyphenols are typically categorized into four main groups: phenolic acids, flavonoids, stilbenes, and lignans. Flavonoids are further subdivided into six principal subclasses: flavan‐3‐ols, flavones, flavonols, flavanones, isoflavones, and anthocyanins. These molecules occur as free aglycones or as sugar‐bound glycosides, the latter being the predominant form in plants (Dias et al. 2021). Additionally, certain flavonoids can undergo polymerization to yield tannins (Rauf et al. 2019).

**TABLE 1:** UPLC‐HRMS data of compounds detected in *S. caseolaris* leaves and roots.

N°	RT (min)	[M − H]^−^	[M + H]^+^	Formula	Error (ppm)	Diagnostic products ion (m/z)	Name	Class	Leaves roots	MSI level^a^	References
1	0.59	377.0851	n.d.	C_18_H_18_O_9_	7.1561	341.1083, 215.0547, 179.0547, 161.0439, 119.0339, 89.0235	Caffeic acid derivative	Caffeic acid derivatives	Roots	2	Riethmüller et al. (2013)
2	0.66	191.0176	n.d.	C_6_H_8_O_7_	9.0719	111.0073, 87.0076, 85.0286	Citric acid	Tricarboxylic acids	Leaves	2	Bylund et al. (2007)
3	0.88	481.0621	n.d.	C_20_H_18_O_14_	0.5783	300.9984, 275.0191	Hexahydroxydiphenoyl‐D‐glucose (HHDP‐glucose)	Ellagitannins	Roots	2	Álvarez‐Fernández et al. (2015)
4	1.15	169.0129	n.d.	C_7_H_6_O_5_	7.9214	125.0228, 97.0279, 81.0338, 79.0182, 69.0341	Gallic acid	Phenolic acids and derivatives	Leaves/Roots	1	std
5	1.66	609.1242	n.d.	C_30_H_26_O_14_	1.2769	441.0821, 423.0715, 305.0652, 177.0175, 125.0228	Prodelphinidin B‐4	Proanthocyanidins	Roots	2	Dou et al. (2007)
6	2.82	305.0650	307.0818	C_15_H_14_O_7_	5.4768	219.0626, 179.0329, 167.0331, 165.0176, 139.0392, 137.0240, 125.0230, 111.0433, 109.0276	(Epi)gallocatechin	Catechins	Leaves/Roots	2	Hofmann et al. (2016)
7	3.44	439.0541	n.d.			241.0011, 197.0446, 138.9694, 96.9588	Unidentified		Roots		
8	3.78	933.0654	n.d.	C_41_H_26_O_26_		915.0551, 889.0781, 631.0568, 613.0564, 587.0659, 569.0590, 425.0163, 300.9985, 275.0190, 249.0405	Vescalagin/castalagin	Ellagitannins	Roots	2	Sanz et al. (2010)
9	4.29	783.0682	n.d.	C_34_H_24_O_22_	0.5688	481.0624, 300.9964, 275.0209	Bis‐HHDP‐glucose (pedunculagin)	Ellagitannins	Leaves	2	Álvarez‐Fernández et al. (2015)
10	4.67	289.0702	291.0868	C_15_H_14_O_6_	5.3837	271.0594, 245.0807, 221.0800, 205.0490, 203.0700, 187.0383, 179.0334, 161.0589, 151.0387, 137.0226, 125.0232, 123.0435, 121.0278, 109.0280, 97.0279, 81.0333	(Epi)catechin	Catechins	Leaves/Roots	2	Hofmann et al. (2016)
11	4.79	483.0779	n.d.	C_20_H_20_O_14_	1.2992	331.0640, 313.0548, 271.0447, 211.0234, 169.0122, 125.0216	Di‐*O*‐galloyl‐β‐D‐glucose isomer	Galloannins	Leaves/Roots	2	Hofmann et al. (2016)
12	5.34	483.0779	n.d.	C_20_H_20_O_14_	0.2663	439.0879, 31.0668,313.0563, 287.0767, 271.0457, 211.0234, 169.0130, 125.0231	Di‐O‐galloyl‐ β‐D‐glucose isomer	Gallotannins	Roots	2	Hofmann et al. (2016)
13	5.46	635.0885	n.d.	C_27_H_24_O_18_	0.7664	483.0744, 465.0660, 423.0563, 313.0553, 295.0457, 169.0125, 125.0230	Tri*‐O*‐galloyl‐β‐D‐glucose isomer	Gallotannins	Leaves	2	Hofmann et al. (2016)
14	5.58	453.1028	n.d.	C_20_H_22_O_12_	2.3115	327.0717, 313.0556, 297.0613, 285.0607, 255.0396, 183.0297, 169.0129, 151.0025, 139.0389, 125.0230, 124.0152	Unidentified	Unidentified gallic acid derivative	Roots	3	—
15	5.69	785.0828	n.d.	C_34_H_26_O_22_	1.9031	633.0743, 615.0633, 483.0741, 419.0607, 300.9970, 275.0790, 249.0380, 169.0117	Di‐*O*‐galloyl‐HHDP‐glucose (tellimagrandin I) isomer	Ellagitannins	Leaves	2	Hofmann et al. (2016)
16	5.89	483.1137	n.d.	C_21_H_24_O_13_	1.4757	327.0713, 313.0548, 297.0615, 285.0594, 183.0294, 169.0134, 155.0329, 140.0101, 125.0225, 124.0149	Unidentified	Unidentified gallic acid derivative	Roots	3	—
17	5.92	467.0834	n.d.	C_20_H_20_O_13_	−0.6106	423.0926, 315.0708, 169.0120, 152.0095, 125.0223	Digalloyl deoxyhexoside	Gallotannins	Leaves/Roots	2	Li and Seeram (2018)
18	6.24	785.0841	n.d.	C_34_H_26_O_22_	0.2493	633.0734, 615.0637, 483.0774, 419.0612, 300.9973, 275.0183, 249.0387, 169.0125	Di‐*O*‐galloyl‐HHDP‐glucose (tellimagrandin I) isomer	Ellagitannins	Leaves	2	Hofmann et al. (2016)
19	6.37	635.0889	n.d.	C_27_H_24_O_18_	0.1376	483.0771, 465.0732, 313.0608, 169.0145	Tri*‐O*‐galloyl‐β‐D‐glucose isomer	Gallotannins	Leaves	2	Hofmann et al. (2016)
20	6.38	457.0773	n.d.	C_22_H_18_O_11_	0.7311	305.0654, 169.0128, 125.0230	(Epi)gallocatechin gallate	Catechins	Roots	2	Dou et al. (2007)
21	6.54	197.0438	n.d.	C_9_H_10_O_5_	8.8209	169.0127, 125.0223, 124.0150	Ethyl gallate	Gallotannins	Leaves/Roots	2	Sun et al. (2007)
22	6.74	445.1339	n.d.	C_19_H_26_O_12_	2.8013	293.1236, 271.0449, 169.0134, 131.0705	Unidentified	Unidentified gallic acid derivative	Roots	3	—
23	6.77	593.1505	595.1658	C_27_H_30_O_15_	1.1677	503.1194, 473.1079, 383.0765, 353.0651	Apigenin 6,8‐di‐*C*‐D‐glucoside (vicenin2)	Flavonoid glycosides	Leaves	2	Hofmann et al. (2016)
24	6.97	523.1447	n.d.	C_24_H_28_O_13_	1.9355	313.0545, 169.0122, 125.0233	Unidentified	Unidentified gallic acid derivative	Leaves	3	—
25	7.20	495.1506	n.d.	C_23_H_28_O_12_	0.4029	313.0541, 181.0853, 169.0137	Unidentified	Unidentified gallic acid derivative	Roots	3	—
26	7.22	447.0922	449.1078	C_21_H_20_O_11_	2.4213	429.0834, 357.0494, 327.0494, 297.0392, 285.0386	Luteolin‐6‐*C*‐glucoside (isorientin)	Flavonoid glycosides	Leaves	2	Sánchez‐Rabaneda et al. (2003)
27	7.39	463.0869	n.d.	C_21_H_20_O_12_	2.8003	301.0335, 300.0256	Quercetin‐3‐*O*‐galactoside (hyperoside)	Flavonoid‐glycosides	Leaves	2	Sánchez‐Rabaneda et al. (2003)
28	7.55	457.1157				260.0346, 96.9588	Unidentified		Roots		—
29	7.65	441.0818	n.d.	C_22_H_18_O_10_	2.0817	289.0699, 245.0821, 169.0125, 125.0223	(Epi)catechin 3‐*O*‐gallate	Catechins	Leaves	2	Dou et al. (2007)
30	7.74	537.1973	n.d.	C_26_H_34_O_12_	0.8363	313.0548, 271.0446, 211.0238, 169.0129, 151.0026, 124.0150	Unidentified	Unidentified gallic acid derivative	Roots	3	—
31	7.85	431.0967	n.d.	C_21_H_20_O_10_	3.8657	341.0642, 323.0529, 311.0539, 283.0589	Apigenin 8‐*C*‐β‐D‐glucoside (vitexin) isomer	Flavonoid glycosides	Leaves	2	Sun et al. (2013)
32	7.91	787.1000	n.d.	C_34_H_28_O_22_	−0.0684	635.0889, 617.0764, 465.0671, 447.0549, 295.0445, 169.0112	Tetra‐*O*‐galloyl‐β‐D‐glucose	Gallotannins	Leaves	2	Hofmann et al. (2016)
33	8.11	463.0869	n.d.	C_21_H_20_O_12_	2.8003	301.0327, 300.0255	Quercetin‐3‐*O*‐glucoside (isoquercitrin)	Flavonoid‐glycosides	Leaves	2	Sánchez‐Rabaneda et al. (2003)
34	8.11	431.0971	433.1135	C_21_H_20_O_10_	2.9400	413.0868, 353.0652, 341.0647, 323.0551, 311.0543, 283.0589, 269.0436	Apigenin 6‐*C*‐β‐D‐glucoside (isovitexin) isomer	Flavonoid glycosides	Leaves	2	Sun et al. (2013)
35	8.34	593.1509	n.d.	C_27_H_30_O_15_	0.4945	285.0386	kaempferol 3‐O‐rutinoside	Flavonoid‐diglycosides	Leaves	2	Sánchez‐Rabaneda et al. (2003)
36	8.37	447.0924	449.1082	C_21_H_20_O_11_	1.9750	285.0394	Luteolin 7‐*O*‐glucoside	Flavonoid‐glycosides	Leaves	2	Sánchez‐Rabaneda et al. (2003)
37	8.40	300.9975	n.d.	C_14_H_6_O_8_	4.9360	283.9957, 257.0080, 229.0130, 185.0232	Ellagic acid	Phenolic acids and derivatives	Roots	1	std
38	8.40	421.1135	n.d.	C_20_H_22_O_10_	1.2331	313.0557, 169.0124, 151.0024, 125.0222	Benzyl‐*O*‐galloylglucose	Gallotannins	Leaves/Roots	2	Ghareeb et al. (2019)
39	8.75	447.0924	n.d.	C_21_H_20_O_11_	1.9750	301.0334, 300.0261	Quercitrin (quercetin 3‐O‐rhamnoside)	Flavonoid‐glycosides	Leaves	2	Sánchez‐Rabaneda et al. (2003)
40	8.92	394.9706	n.d.	C_15_H_8_O_11_S	2.1607	315.0128, 299.9896	Isorhamnetin sulfate	Flavonoid derivatives	Roots	2	Su et al. (2010)
41	8.92	315.0137	317.0291	C_15_H_8_O_8_	3.2934	299.9898, 282.9861, 270.9882, 243.9994, 228.0048, 216.0054, 200.0098, 172.0157, 160.0153	3‐O‐methylellagic acid	Phenolic acid and derivatives	Roots	2	Rosa et al. (2021)
42	9.26	431.0982	n.d.	C_21_H_20_O_10_	0.3943	269.0427, 268.0358	Genistein 7‐O‐glucoside (genistin)	Isoflavonoid‐glycosides	Leaves	2	Li et al. (2012)
43	9.73	409.0199	n.d.	C_17_H_14_O_10_S	−7.4813	329.0294, 314.0059, 298.9823	Quercetin dimethyl ether sulfate	Flavones derivatives	Roots	2	Taamalli et al. (2015)
44	10.09	285.0385	n.d.	C_15_H_10_O_6_	6.8576	257.0439, 243.0291, 241.0497, 217.0485, 199.0385, 175.0380, 151.0017, 133.0276, 107.0117	Luteolin	Flavones	Leaves	1	std
45	10.24	461.0718	n.d.	C_21_H_18_O_12_	1.6222	315.0134, 299.9897	Isorhamnetin pentoside	Flavonoid‐glycosides	Leaves/Roots	2	Hofmann et al. (2016)

Abbreviations: n.d., not detected; std, standard compound.

^a^ According to metabolomics standards initiative (MSI).

Analytes were characterized by the combination of accurate mass measurements, retention times, UV–Vis absorbance profiles, and MS/MS fragmentation spectra, with reference to the literature databases and standard compounds where available.

#### 3.2.1 Phenolic Acid and Derivatives

Four phenolic acid compounds were detected. These include two phenolic acids in their underivatized forms: gallic acid (**4**) and ellagic acid (**37**), with [M–H]^−^ ions at *m/z* 169.0129 (C_7_H_6_O_5_) and 300.9975 (C_14_H_6_O_8_), respectively. These molecules were identified by a coincidence of the MS fragmentation pattern with previous literature data (Lee et al. 2005) and confirmed by reference standards. Peak **1** showed a [M–H]^−^ ion at *m/z* 377.0851 (C_18_H_18_O_9_) and was assigned to a caffeic acid derivative (Riethmüller et al. 2013). The product ions observed during fragmentation were *m/z* 215.0547 [M–H–162]^−^ (loss of a hexose unit), 179.0547 [caffeic acid–H]^−^, and 161.0439 [caffeic acid–H–H_2_O]^−^. Peak **41**, with [M–H]^−^ ion at *m/z* 315.0137 (C_15_H_8_O_8_), was assigned to 3‐*O*‐methylellagic acid based on the coincident MS fragmentation pattern with previous literature data (Rosa et al. 2021).

Among the tentatively identified phenolic acids, four were detected in roots, and among these, only gallic acid was also present in leaves. This pattern likely reflects their defensive role against soil pathogens (Mandal et al. 2010; Pratyusha 2022; Saini et al. 2024). Both gallic acid (4) and ellagic acid (37) have been previously identified in *S. caseolaris*, with gallic acid detected in leaves and ellagic acid found in leaves and fruits, while 3‐O‐methylellagic acid (41) has only been previously identified before in the mangrove *Lumnitzera racemosa* (Fang et al. 2019; Phuong et al. 2017).

#### 3.2.2 Flavonoids

##### 3.2.2.1 Flavonoids Glycosides

Most flavonoids are found in glycosylated form, as glycosylation improves their solubility, transport, and metabolism. In the present study, 11 flavonoid glycosides and derivatives were identified in *S. caseolaris* extracts, almost exclusively in leaf extract, in agreement with previous findings (Cerri et al. 2025) and with their well‐known function as antioxidants protecting against UV radiation and other abiotic stresses (Di Ferdinando et al. 2011). The most abundant glycosides are O‐linked, in which the sugar moiety is bound via hydroxylic oxygen, although C‐linked variants, characterized by direct carbon–carbon bonds between sugar and flavonoid core, are also common (Dias et al. 2021; Xie et al. 2022). The two types of glycosylation exhibit distinct fragmentation patterns. C‐glycosides produce neutral MS/MS losses of 30, 90 and 120 Da for hexose sugars, 74 and 104 Da for deoxyhexose sugars, 60 Da for pentose sugars, and 18 Da for water loss (Hofmann et al. 2016). The peak **26**, with [M–H]^−^ ion at *m/z* 447.0922 (C_21_H_20_O_11_), was assigned to luteolin‐6‐C‐glucoside (isorientin). Fragments ions were detected at *m/z* 429.0834 [M–H–18]^−^ (loss of H_2_O), 357.0494 [M–H–90]^−^ and 327.0494 [M–H–120]^−^ (neutral loss of C‐hexoside), and 297.0392 [M–H–150]^−^ (Sánchez‐Rabaneda et al. 2003). The ion at *m/z* 285.0386 corresponds to luteolin. Following the same fragmentation pathway the peak **23**, **31**, **34** were attributed to apigenin 6,8‐di‐C‐D‐glucoside (vicenin 2), 8‐C‐glycosidic flavonoid (vitexin) and 6‐C‐glycosidic flavonoid (isovitexin), respectively. The O‐glycosides typically involve neutral losses of 162 Da for hexose, 146 Da for deoxy‐hexose, and 132 Da for pentose sugars. Therefore peak **36**, with a [M–H]^−^ ion at *m/z* 447.0924 (C_21_H_2_O_11_) and a *m/z* 285.0394 [M–H–162]^−^ ion produced in the fragmentation spectrum by the neutral loss of an O‐hexoside, corresponded to luteolin 7‐*O*‐glucoside (Hofmann et al. 2016). Based on this fragmentation pathway the peak **27**, **33**, **35**, **39**, **42**, **45** were tentatively identified as quercetin‐3‐*O*‐galactoside (hyperoside), quercetin‐3‐*O*‐glucoside (isoquercitrin), kaempferol 3‐*O*‐rutinoside, quercetin 3‐*O*‐rhamnoside (quercitrin), genistein 7‐*O*‐glucoside (genistin) and isorhamnetin‐*O*‐pentoside. In addition, peak **44** was detected as the free aglycon luteolin with its a [M–H]^−^ ion at *m/z* 285.0385 (C_15_H_10_O_6_) by a coincident MS fragmentation pattern with data from previous literature (Sánchez‐Rabaneda et al. 2003) and confirmed by analytical standard.

Regarding the flavonoid glycosides identified in the present study, only isovitexin (**34**), quercitrin (**39**), and luteolin 7‐glucoside (**36**) have previously been reported in *S. caseolaris* (Audah et al. 2022; Kundu et al. 2023). Isorientin (**26**), hyperoside (**27**), vitexin (**31**), isoquercitrin (**33**), and kaempferol 3‐*O*‐rutinoside (**35**) have been reported in other mangrove species (Chiavaroli et al. 2020; Vinh et al. 2021; Glasenapp et al. 2019) but not in *S. caseolaris*. Furthermore, apigenin 6,8‐d‐C‐D‐glucoside (**23**), genistein 7‐*O*‐glucoside (**42**), and isorhamnetin‐*O*‐pentoside (**45**) have not been identified in mangroves prior to this study.

Flavonoids represented one of the major classes of secondary metabolites identified in this study. This finding is consistent with previous work showing that flavonoid glycosides are abundantly present in leaf extracts of *S. caseolaris* from other geographic regions (Cerri and Galli 2025). A similar pattern has been reported for other mangrove species growing in extreme habitats (Cerri et al. 2025). Together, these observations highlight the prominent role of flavonoids in mangrove adaptation, as these compounds are widely associated with tolerance to abiotic stresses that are characteristic of mangrove environments.

##### 3.2.2.2 Flavonoid Derivatives

Two sulfated flavonoids were detected in the root extract. Peak **40**, with a [M–H]^−^ ion at *m/z* 394.9706 (C_15_H_8_O_11_S), was classified as isorhamnetin sulfate, evidenced by its product ion at *m/z* 315.0128 corresponding to the loss of an 80 Da sulfate moiety, along with the ion at *m/z* 299.9896 (Su et al. 2010). Peak **43**, a [M–H]^−^ ion at *m/z* 408.9863 (C_17_H_14_O_10_S) and fragment ions at *m/z* 329.0294 (quercetin dimethyl ether ion), 314.0059, and 298.9823, was tentatively identified as quercetin dimethyl sulfate (Taamalli et al. 2015).

Sulfated flavonoids have been increasingly reported in plants occurring in waterlogged or mineral‐rich environments, including some saline habitats, suggesting that sulfate conjugation may represent a relevant biochemical response to such conditions. Sulfation has been proposed to facilitate the detoxification or compartmentalization of excess inorganic sulfate, contributing to the maintenance of ionic balance and the mitigation of oxidative stress. Usually, their occurrence has been reported in several halophytic and marine species such as (*Myriophyllum, Zostera*, *and Halophila*), where sulfated flavonoids are attributed to the high concentration of sulfate ions in seawater, providing valuable insights into their possible functions (Teles et al. 2018). The detection of two sulfated flavonoids in the roots of *S. caseolaris*, reported here for the first time in this species, suggests that sulfate conjugation may represent a habitat‐specific adaptive response to the swampy, waterlogged conditions of the inland marsh mangrove where the plants were sampled. However, the exact functional role of flavonoids in plant cells is not yet fully understood.

In addition to these ecological implications, sulfated flavonoids have attracted interest for their biological activities, as sulfation can modify the pharmacological profile of the parent flavonoid by altering solubility, stability, and interactions with molecular targets. Reported activities include anticoagulant, anti‐inflammatory, antimicrobial, antiviral, anticancer, antioxidant, and antidiabetic effects, in some cases differing from those of the corresponding nonsulfated analogs (Teles et al. 2018; Mohammed et al. 2025).

#### 3.2.3 Catechins

Catechins, polyphenolic flavonoids classified as flavan‐3‐ols (or flavanols), possess potent antioxidant properties (Bedlack et al. 2015). Four catechins were detected in *S. caseolaris* extracts.

Peak **10**, with a [M–H]^−^ ion at *m*/*z* 289.0702 (C_15_H_14_O_6_), corresponded to (epi)catechin based on its distinctive fragment ions at *m/z* 245.0807 [M–H–CO_2_]^−^, 205.0490 [M–H–2C_2_H_2_O]^−^, 203.0700 [M–H–CO_2_–C_2_H_2_O]^−^, and 137.0226 [M–H–C_8_H_8_O_3_]. The [M − H]^−^ ion resulted in several other fragment ions, some of which were reported in the MS fragmentation of previous studies, including ions at *m/z* 271.0594, 221.0800, 179.0334, 125.0232, 109.0280 (Hofmann et al. 2016).

Peak **6**, with a [M–H]^−^ ion at *m/z* 305.0650 (C_15_H_14_O_7_), was attributed to (epi)gallocatechin based on its MS fragmentation pattern, which matched previously reported data (Hofmann et al. 2016). The product ions observed during fragmentation were *m/z* 219.0262 [M–H–C_4_H_6_O_2_]^−^, 179.0329 [M–H–C_6_H_6_O_3_]^−^, 167.0331 [M–H–C_7_H_6_O_3_]^−^, and 165.0176 [M–H–C_7_H_8_O_3_]^−^. The fragment ion at *m/z* 125.0230 corresponds to the intact flavonoid A ring (Pacifico et al. 2014).

Peak **20**, with a [M–H]^−^ ion at *m/z* 457.0773 (C_27_H_18_O_11_), was assigned to (epi)gallocatechin gallate with characteristic fragments at *m/z* 305.0654 and 169.0128, distinctive deprotonated ions of (epi)gallocatechin and gallic acid, respectively (Pacifico et al. 2014).

Peak **29**, with a [M–H]^−^ ion at *m/z* 441.0818 (C_22_H_18_O_10_), was attributed to (epi)catechin‐3‐O‐gallate (Dou et al. 2007). The detection of neutral loss of 152 Da, indicative of a galloyl unit, alongside the presence of a (epi)catechin moiety was confirmed by distinctive fragment ions at *m/z* 289.0699 [M–H–152]^−^ and 245.0821 [M–H–152–44]^−^ (loss of CO_2_). Furthermore, the ions at *m/z* 169.0125 and 125.0223 are the diagnostic ions of a galloyl moiety.

Among these, only epigallocatechin gallate (**20**) has been previously reported in *S. caseolaris* (Dahibhate et al. 2021). (Epi)catechin (**10**) and (Epi)gallocatechin (**6**) have been identified in various species of mangroves, including *Aegiceras corniculatum*, *Rhizophora* spp., and *L. racemosa* (Glasenapp et al. 2019; Wei et al. 2012; Rahim et al. 2008). However, to our knowledge, this is the first report of these flavonoids in *S. caseolaris*. Additionally, (epi)catechin‐3‐*O*‐gallate (**29**) has previously been found in *A. corniculatum*, but not in *S. caseolaris* (Wei et al. 2012; Rahim et al. 2008).

#### 3.2.4 Tannins

Tannins consist of multiple units with polyhydroxyphenolic groups or their derivatives, capable of forming complexes with other substances such as proteins, cellulose, and minerals. Tannins can be broadly categorized into hydrolysable and condensed tannins. Esters of gallic acid and polyols, primarily D‐glucose, constitute the hydrolysable tannin group, which is further classified into gallotannins or ellagitannins depending on whether they yield gallic acid or ellagic acid upon hydrolysis. Condensed tannins, referred to as proanthocyanidins, are oligomeric structures composed of flavonoid subunits that vary in degree of polymerization (Rauf et al. 2019). Tannins are well‐documented antioxidant metabolites (Koleckar et al. 2008) and were similarly distributed between leaf and root extracts in this study. Among them, gallotannins were the most abundant and are recognized for their role in plant defense against herbivores and pathogens (He 2022).

##### 3.2.4.1 Gallotannins

Eight gallotannins were detected in the present study. Peaks **11** and **12** were assigned to two di‐O‐galloyl‐D‐glucose isomers, with a [M–H]^−^ ion at *m/z* 483.0779 (C_20_H_20_O_14_). The MS2 spectra for both isomers revealed distinctive fragments at *m/z* 331.0640, 313.0548, 271.0447, 211.0234, 169.0122 and 125.0216, consistent with data from the literature (Hofmann et al. 2016). Ions at *m/z* 331 and 313 were formed due to the elimination of units of galloyl (152 Da) and gallic acid (170 Da), respectively, and are indicative of the structural class of gallotannin. The successive loss of C_2_H_2_O, CH_2_O and CH_2_O from the ion at *m/z* 313 produced ions at *m/z* 313 produced ions 271, 241 and 211, respectively. Additional key diagnostic ions included *m/*z 169 [gallic acid–H]^−^ and 125 [gallic acid–H–44]^−^ (loss of CO_2_). Gallotannins are often present with varying pattern of galloyl substitution. Repeated neutral loss events at *m/z* 152 corresponding to galloyl moieties. On this basis, peaks **13** and **19** with a [M–H]^−^ ion at *m/z* 635.0885 (C_27_H_24_O_18_) were assigned to tri‐*O*‐galloyl‐D‐glucose, while peak **32** with a [M–H]^−^ ion at *m/z* 787.1000 (C_34_H_28_O_22_) was attributed to tetra‐*O*‐galloyl‐β‐D‐glucose (Hofmann et al. 2016) and peak **17**, with a [M–H]^−^ ion at *m/z* 467.0834 (C_20_H_20_O_12_), corresponded to luteolin digalloyl deoxyhexoside (Li and Seeram 2018), peak **21**, with a [M–H]^−^ ion at *m/z* 197.0438 (C_9_H_10_O_5_), was assigned to ethyl gallate, an ester of gallic acid and ethanol (Sun et al. 2007), and peak **38**, with a [M–H]^−^ ion at *m/z* 421.1135 (C_20_H_22_O_10_), corresponded to benzyl‐*O*‐galloylglucose (Ghareeb et al. 2019).

Tri‐*O*‐galloyl‐β‐D‐glucose (**13**, **19**), ethyl gallate (**21**), and tetra‐*O*‐galloyl‐β‐D‐glucose (**32**) have been previously found in other mangrove species (Li et al. 2012). However, to our knowledge, di‐*O*‐galloyl‐D‐glucose isomers (**11**, **12**) and benzyl‐*O*‐galloylglucose (**38**) have not previously been reported from mangroves. Furthermore, none of these compounds have been reported in *S. caseolaris*.

##### 3.2.4.2 Ellagitannins

Five ellagitannins were identified in the present study. The diagnostic fragment ion of this class of compounds is *m/z* 301 [ellagic acid–H]^−^, and the loss of the HHDP (hexahydroxydiphenoyl) groups, resulting in *m/z* [M–H–302]^−^ fragments (Hofmann et al. 2016). On the basis of this pattern of fragmentation, the peaks **3**, **8**, **9**, **15**, and **18** were tentatively identified as hexahydroxydiphenoyl‐D‐glucose (HHDP‐glucose), vescalagin/castalagin, bis‐HHDP‐glucose (pedunculagin), and di‐*O*‐galloyl‐HHDP‐glucose (tellimagrandin I) isomers 1 and 2. HDPP‐glucose (**3**), pedunculagin (**8**), and tellimagrandin I (**15**, **18**) have not previously been reported from mangrove species.

##### 3.2.4.3 Proanthocyanidins (Condensed Tannins)

Peak **5**, with a [M–H]^−^ ion at *m/z* 609.1242 (C_30_H_26_O_14_), was identified as prodelphinidin B‐4 (Riethmüller et al. 2013), a (epi)gallocatechin‐(4,8′)‐(epi)gallocatechin dimer (Jaiswal et al. 2012), never before identified from mangroves. In fact, except the ion at *m/z* 305.0652, in accordance with the cleavage of epigallocatechin (or gallocatechin), the fragment ions at *m/z* 441.0821 [M–H–168]^−^ and 423.0715 [M–H–168–18]^−^ were produced via a retro–Diels–Alder (RDA) cleavage pattern involving the C‐ring, particularly breaking the O1–C2 and C3–C4 bounds, followed by dehydration (Pacifico et al. 2014).

Notably, we detected a total of 14 tannins, making tannins one of the major chemical classes identified in this study. To date, only four tannins have been previously reported from *S. caseolaris* (Cerri and Galli 2025). This discrepancy likely reflects differences in tissue coverage, because earlier studies focused on other parts of *S. caseolaris*, even though mangrove roots are often particularly rich in tannins, where they may contribute to stress mitigation and microbial interactions in the rhizosphere (Kimura and Wada 1989). Tannins are also abundant constituents of leaves, where they function primarily in defense against herbivores and pathogens (He 2022; Constabel et al. 2014). Nevertheless, foliar tannins of *S. caseolaris* have likewise been only marginally explored to date (Cerri and Galli 2025). The comparatively high tannin diversity observed here may therefore reflect both previous under‐sampling of roots and leaves and ecological features of Maldivian habitats, including potential pressure from herbivores and soil pathogens. In addition, tannins may participate in tolerance to abiotic stress, although their precise roles in stress adaptation remain insufficiently resolved and warrant further investigation (Constabel et al. 2014; Iqbal and Poór 2025).

#### 3.2.5 Nonpolyphenolic Compounds

Peak **2**, with a [M–H]^−^ ion at *m/*z 191.0176 (C_6_H_8_O_7_), was assigned to citric acid. Identification was based on its coincident pattern of MS fragmentation with previous literature data (Bylund et al. 2007).

#### 3.2.6 Unknown Gallic Compounds

Peak **14**, with a [M–H]^−^ ion at *m/z* 453.1028 (C_20_H_22_O_12_), was classified as an unidentified gallic acid derivative for the diagnostic fragment ions at *m/z* 169.0129 [gallic acid–H]^−^, 151.0025 [galloyl–H]^−^, and 125.0230 [gallic acid–H–44]^−^. Although the fragmentation behavior resembles that reported for galloylquercetin (Hofmann et al. 2016), the absence of characteristic quercetin fragments (*m/z* 301, 300) and the presence of a fragment at *m/z* 285.0607 (luteolin or kaempferol) suggest an alternative structure.

Peak **16**, with a [M–H]^−^ ion at *m/z* 483.1137 (C_21_H_24_O_13_), exhibited a comparable MS/MS fragmentation profile to that of **14**. It was classified as an unidentified gallic acid derivative, with diagnostic fragment ions at *m/*z 169.0134 [gallic acid–H]^−^ and 125.0225 [gallic acid–H–44]^−^.

Peak **22**, with a [M–H]^−^ ion at *m/z* 445.1339 (C_19_H_26_O_12_), was classified as an unidentified gallic acid derivative for the characteristic product ions observed during fragmentation: *m/z* 293.1236 [M–H–galloyl]^−^ and 169.0134 [gallic acid–H]^−^.

Peak **24**, with a [M–H]^−^ ion at *m/z* 523.1447 (C_24_H_28_O_13_), peak **25**, with a [M − H] − ion at *m/z* 495.1506 (C_24_H_28_O_12_), and peak **30**, with a [M − H] − ion at *m/z* 537.1973 (C_26_H_34_O_12_), were similarly classified as unidentified gallic acid derivatives for the characteristic key ion *m/*z 169.0122 [gallic acid–H]^−^ produced in the fragmentation spectrum. Furthermore, fragment ions at *m/z* 125.0233 [gallic acid–H–44]^−^ and 151.0026 [galloyl−H] − were detected for peaks **24** and **30**, respectively.

Although the precise structures of these compounds could not be fully resolved, their occurrence may be biologically relevant because gallic acid‐based metabolites are widely associated with bioactive potential. Gallic acid derivatives have been reported to be antioxidant, antimicrobial, antiviral, anticancer, anti‐inflammatory, and neuroprotective (Lu et al. 2006; Badhani et al. 2015).

### 3.3 Antioxidant Activity

**FIGURE 5:**
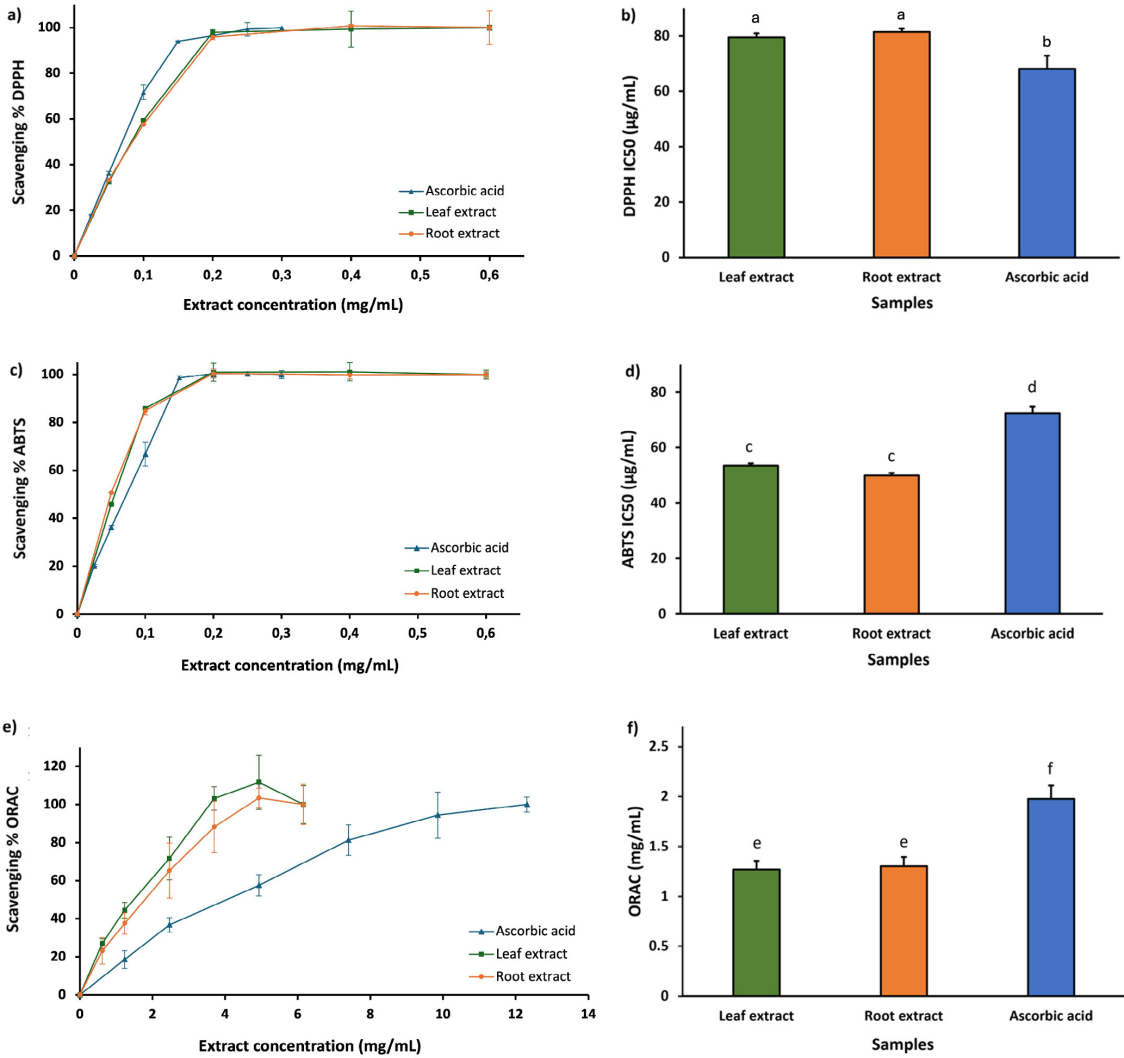
Antioxidant activities of *Sonneratia caseolaris* leaf (green) and root (orange) extract species determined by DPPH, ABTS, and ORAC assays. Ascorbic acid was used as a positive control (blue). The graphic (a), (c), and (e) show the dose‐dependent response of samples and positive control in DPPH, ABTS, and ORAC assays, respectively. The graphic (b), (d), and (f) compare the IC_50_ of samples and positive control. All measurements were performed in triplicate (*n* = 3). Different letters in each graph indicate a statistical difference (*p*‐value < 0.05).

Because of the abundant presence of secondary metabolites known for their antioxidant power, such as phenolic compounds, the radical scavenging capacity was monitored using spectrophotometric assays. The activity of leaf and root extracts was investigated by DPPH, ABTS, and ORAC assays (Figure 5). The results of all assays showed a concentration‐dependent antioxidant effect (Figure 5a,c,e). The antioxidant activity was expressed as IC_50_, where a lower IC_50_ indicates a higher free radical scavenging activity. In detail, in the DPPH assay the IC_50_ values for leaf and root extracts were 79.55 ± 1.32 and 81.40 ± 1.22 μg/mL, respectively, demonstrating slightly lower antioxidant activity than ascorbic acid (IC_50_: 68.15 ± 4.71 μg/mL) (Figure 5b). Instead, the ABTS test reported IC_50_ values of 53.53 ± 0.79 μg/mL (leaf) and 49.95 ± 0.90 μg/mL (root), significantly lower than those of ascorbic acid (IC_50_: 72.40 ± 2.34 μg/mL) (Figure 5d). The ORAC assay further validated the potent antioxidant properties of hydroalcoholic extracts with IC_50_ values of 1.267 ± 0.088 (leaf) and 1.302 ± 0.092 μg/mL (root), lower than the positive control (IC_50_ 1.975 ± 0.134 μg/mL). No statistically significant differences between leaves and roots were found, indicating that they are equally effective in neutralizing free radicals. The present results are consistent with literature values for the anti‐scavenger effect of mangrove. Reported DPPH IC_50_ for *S. caseolaris* leaf extracts span a broad range (1.92–171 μg/mL), and our results fall within the middle of this interval (Cerri and Galli 2025). This emphasizes the high anti‐scavenger power of *S. caseolaris* plants grown in the Maldives, which is consistent with observations made on other plants of the same species. In addition, the ABTS IC_50_ values obtained here are comparable to those reported for ellagitannin‐rich *S. caseolaris* leaf fractions (Fang et al. 2019). Furthermore, the antioxidant power observed in *S. caseolaris* is higher or comparable to that observed in other mangroves. A study of *Avicennia marina* , one of the best known and most studied mangroves, showed an antioxidant capacity in the ethanolic extract of the leaves with IC_50_ 257.04 ± 3.30 μg/mL (DPPH) and 42.73 ± 0.36 μg/mL (ABTS) (Nguyen et al. 2022).

*S. caseolaris* has been shown to be very effective against various radicals. This demonstrates its promise as a natural antioxidant reservoir. However, it should be noted that, although the three spectrophotometric assays used here are widely applied for evaluating antioxidant activity in mangrove extracts (Thatoi et al. 2014), they provide only a limited scope, serving primarily as indicators of the plant's capacity to neutralize free radicals. Additional in vitro studies, as well as cell‐based and in vivo oxidative stress models, will be required to fully clarify the functional relevance and mechanisms of action of the detected metabolites.

## 4 Conclusions

This research constitutes the first in‐depth investigation into the phytochemical composition and antioxidant behavior of S. caseolaris leaves and roots collected in the Maldives. Using UPLC‐ESI/HRMS analyses, 45 compounds were detected. Among these, 36 were identified as polyphenolic compounds belonging to major categories such as phenolic acids and their derivatives, flavonoids, and tannins. The predominant groups were flavonoid glycosides (11 compounds) and gallotannins (8 compounds). Additionally, six unidentified compounds exhibited characteristic fragmentation patterns of gallic acid derivatives, while six others were classified as nonpolyphenolic compounds. The total number of detected metabolites was comparable between leaves and roots, with 20 compounds exclusive to the leaves, 17 to the roots, and 8 common to both extracts. However, some differences emerged in the dominant chemical classes: flavonoid glycosides were more typical of the leaves, while phenolic acids were relatively more abundant in the roots. Notably, only six of the identified compounds had previously been reported from *S. caseolaris*. Importantly, our analysis also revealed several compounds not previously documented in this species, including two sulfated flavonoids, metabolites commonly associated with plants adapted to swampy and mineral‐rich environments. These observations suggest that the unique Maldivian habitat of *S. caseolaris* may have influenced the biosynthesis of distinctive secondary metabolites.

Both leaf and root extracts demonstrated significant antioxidant capacities in three spectrophotometric assays, highlighting their potential as sources of bioactive compounds. This study offers important perspectives on the chemical and biological properties of *S. caseolaris* and lays the foundation for further investigations into its pharmacological and nutraceutical applications. Given their rich antioxidant profile, *S. caseolaris* extracts could be explored for the development of natural health supplements and cosmetic products with anti‐aging and skin‐protection benefits. Furthermore, its bioactive compounds are promising for therapeutic applications, particularly in the management of disorders related to oxidative stress. Future studies should include preliminary cytotoxicity or acute toxicity evaluations to confirm the safety of these extracts and to further substantiate their nutraceutical and pharmacological potential, as well as in vivo and mechanistic investigations to better elucidate their antioxidant relevance and modes of action. Additionally, future work should also include collections from different locations and seasons to evaluate environmental and phenological influences on the metabolite profile of *S. caseolaris*. Furthermore, to improve the data on this species, fruits and other parts could be included in future sampling to obtain a more complete phytochemical picture of the species in this region. The findings of this study also emphasize the need for further bioprospecting research on underexplored mangrove species to unlock their full therapeutic and commercial potential.

## Author Contributions

**Federico Cerri:** investigation, writing – original draft, data curation. **Stefania Pagliari:** writing – original draft, investigation, formal analysis. **Shazla Mohamed:** investigation, formal analysis, writing – original draft. **Massimo Labra:** conceptualization, funding acquisition, project administration. **Luca Campone:** conceptualization, writing – original draft, funding acquisition, data curation, supervision. **Paolo Galli:** supervision, funding acquisition, conceptualization, project administration.

## Funding

This work was supported by project funded under the National Recovery and Resilience Plan (NRRP), Mission 4. Component 2 Investment 1.3—Call for tender No. 3138, 16 December 2021, rectified by Decree n.341 of 15 March 2022 of the Italian Ministry of University and Research funded by the European Union—NextGenerationEU; Project code PE0000003 ON FOODS—CUP: H43C22000820001—Spoke 6, Project title “ON Foods—Research and innovation network on food and nutrition Sustainability, Safety and Security—Working ON Foods”. National Biodiversity Future Center (NBFC), Palermo Italy (funded by the European Union's Next Generation EU; Project code CN00000033; CUP: H43C22000530001).

## Conflicts of Interest

The authors declare no conflicts of interest.

## Supporting information


**FIGURE S1:** Chromatograms of leaves (A at 280 nm and B at 330 nm) and roots (C at 280 nm and D at 330 nm) with different extraction solvents 100% water (green), 100% EtOH (violet) and 50% EtOH‐water (red).

## Data Availability

The data that support the findings of this study are available on request from the corresponding author. The data are not publicly available due to privacy or ethical restrictions.
