# Supplementary material for: Phytochemical Composition and Antioxidant Activities of Sonneratia caseolaris (L.) Engl. Leaves and Roots: Insights Into a Promising Mangrove Species
Source: Food Sci Nutr. 2026 Apr 18;14(4):e71664. doi: 10.1002/fsn3.71664 (PMC13091725; doi:10.1002/fsn3.71664)
Supplement: Supplementary file 1 — FIGURE S1: Chromatograms of leaves (A at 280 nm and B at 330 nm) and roots (C at 280 nm and D at 330 nm) with different extraction solvents 100% water (green), 100% EtOH (violet) and 50% EtOH‐water (red). [file FSN3-14-e71664-s001.docx]

Figure S1: Chromatograms of leaves (A at 280 nm and B at 330 nm) and roots (C at 280 nm and D at 330 nm) with different extraction solvents 100% water (green), 100% EtOH (violet) and 50% EtOH-water (red)


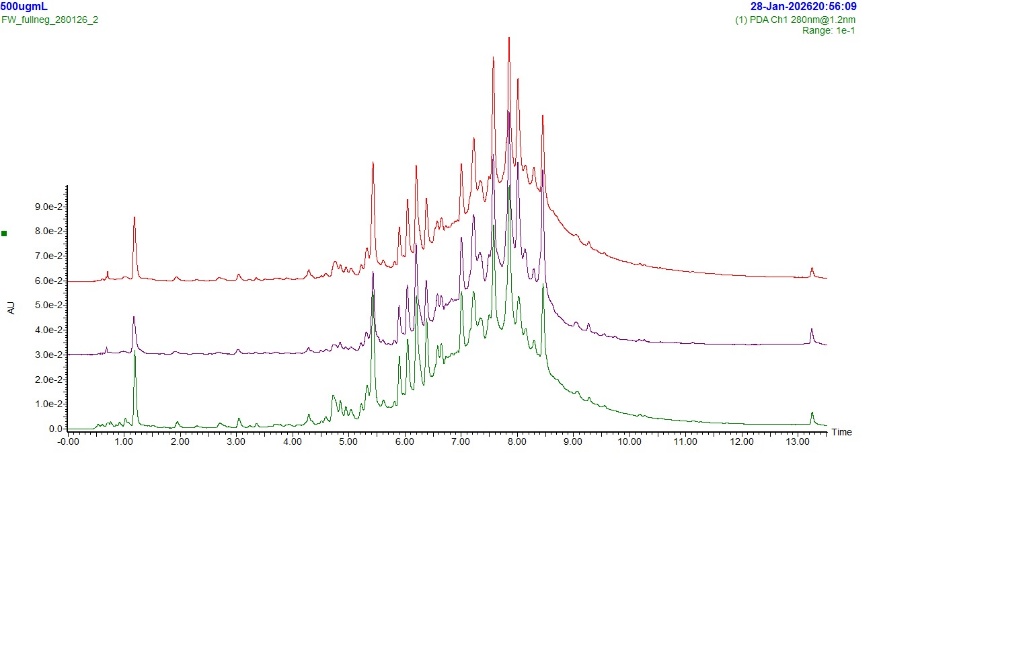


AU

100% EtOH

100% H_2_O

50% EtOH-H_2_O

A

min


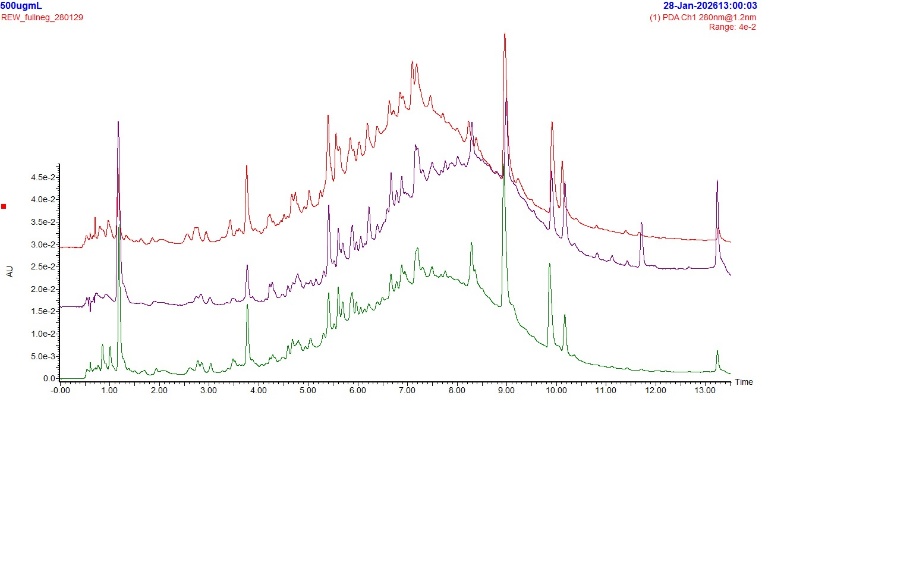


AU

C

100% EtOH

100% H_2_O

50% EtOH-H_2_O

min


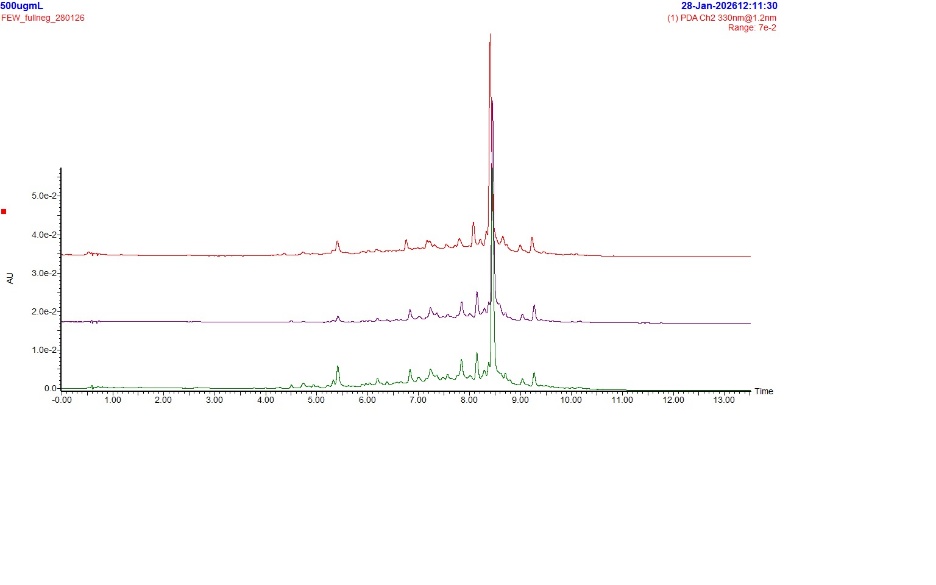


AU

B

100% EtOH

100% H_2_O

50% EtOH-H_2_O

min


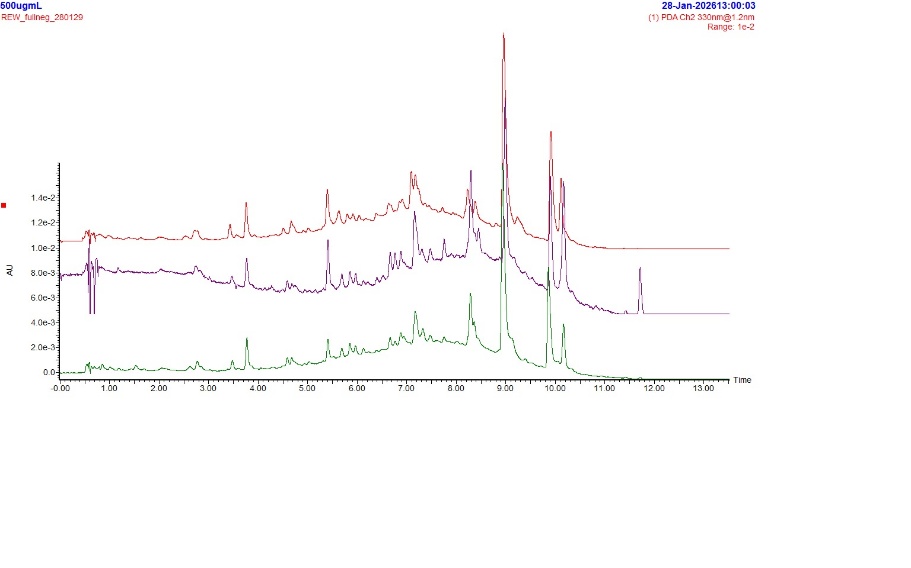


AU

D

100% EtOH

100% H_2_O

50% EtOH-H_2_O

min
